# The Exposure Uncertainty Analysis: The Association between Birth Weight and Trimester Specific Exposure to Particulate Matter (PM_2.5_ vs. PM_10_)

**DOI:** 10.3390/ijerph13090906

**Published:** 2016-09-13

**Authors:** Naresh Kumar

**Affiliations:** Department of Public Health Sciences, University of Miami, Miami, FL 33136, USA; nkumar@med.miami.edu; Tel.: +1-305-243-4854

**Keywords:** exposure uncertainty, spatiotemporal autocorrelation, semivariance, coarse and fine particulates, Chicago, air pollution epidemiology

## Abstract

Often spatiotemporal resolution/scale of environmental and health data do not align. Therefore, researchers compute exposure by interpolation or by aggregating data to coarse spatiotemporal scales. The latter is often preferred because of sparse geographic coverage of environmental monitoring, as interpolation method cannot reliably compute exposure using the small sample of sparse data points. This paper presents a methodology of diagnosing the levels of uncertainty in exposure at a given distance and time interval, and examines the effects of particulate matter (PM) ≤2.5 µm and ≤10 µm in diameter (PM_2.5_ and PM_10_, respectively) on birth weight (BW) and low birth weight (LBW), i.e., birth weight <2500 g in Chicago (IL, USA), accounting for exposure uncertainty. Two important findings emerge from this paper. First, uncertainty in PM exposure increases significantly with the increase in distance from the monitoring stations, e.g., 50.6% and 38.5% uncertainty in PM_10_ and PM_2.5_ exposure respectively for 0.058° (~6.4 km) distance from the monitoring stations. Second, BW was inversely associated with PM_2.5_ exposure, and PM_2.5_ exposure during the first trimester and entire gestation period showed a stronger association with BW than the exposure during the second and third trimesters. But PM_10_ did not show any significant association with BW and LBW. These findings suggest that distance and time intervals need to be chosen with care to compute exposure, and account for the uncertainty to reliably assess the adverse health risks of exposure.

## 1. Introduction

The causal mechanism of the health effects of the environment should remain the same in the same population. For example, we already know that elevated exposure to ambient particulate matter (PM) retards fetal growth due to oxidative stress [1]. This means that the same level of PM exposure must have the same effects on birth weight (BW) and low birth weight (LBW) in the same population. However, observational studies have been inconsistent concerning the risks of adverse birth outcomes associated with PM exposure [2,3]. A recent study by Kumar empirically demonstrates that exposure uncertainty is one of the important reasons underlying inconsistency in the adverse effects of criteria pollutants, including PM, CO, SO_2_, O_3_ and NO_2_. Sparse spatiotemporal monitoring, mismatch in the spatiotemporal scale/resolution and spatiotemporal misalignment of environment and health data set are one of the main reasons for exposure uncertainty [4]. Generally, researchers compute exposure by interpolation techniques or by aggregating data to coarse geographic and temporal scales. The latter is often a preferred choice in the epidemiological studies because of sparse geographic coverage of environmental monitoring that restricts the scope of interpolation methods to reliably compute exposure. For example, researchers average the values from all monitoring stations within a county to compute exposure of all subjects within that county (on the same day) [5]. However, the Environmental Protect Agency (EPA) data suggests that the range of PM ≤ 10 µm in aerodynamic diameter (PM_10_) can be as high as 40 µg/m^3^ within a city on a given day [6,7]. Thus, the use of coarse distance intervals (or by county level aggregation) and ad-hoc methods of interpolation are likely to result in exposure uncertainty and potentially exposure misclassification, e.g., if there are two PM monitoring sites in a county and these sites report 8 µg/m^3^ and 16 µg/m^3^ concentration of fine PM ≤ 2.5 µm in aerodynamic diameter (PM_2.5_) respectively on a given day, aggregating these data will result in an average daily exposure of 12 µg/m^3^ for the county, resulting in exposure misclassification of individuals living at/around these sites, and generalization (by computing average) for the entire county by losing the precise data at/around these sites. This paper advances the previous research on exposure uncertainty [8] by presenting a novel method of diagnosing exposure uncertainty and examining the effects of trimester specific PM exposure on BW and LBW in Chicago Metropolitan Statistical Area (Chicago from here onward) between 2000 and 2004 accounting for exposure uncertainty.

The two hypotheses of this paper are derived by spatiotemporal heterogeneity in PM and differential biophysiological impacts of different particle sizes. First, it is hypothesized that exposure uncertainty increases as the distance and time interval from the monitored data (or true measurement) increases, because there are subtle spatiotemporal variations in fine and coarse particulate matter (PM) largely because of spatial heterogeneity in air pollution sources and air pollution transport. Moreover, the coarse particle settles with gravity quicker than the fine particles [9], it is further hypothesized that the exposure uncertainty for PM_10_ (coarse particles) will be greater than that for PM_2.5_ as the distance intervals to monitoring station(s) increases. Although PM exposure can occur at home, work, indoors, outdoors and while commuting, this retrospective study relied on ambient PM data, and exposure was computed at the census tract of residence. Because time-activity of subjects and indoor PM data at work and home were not available. From here onward, “PM exposure” will refer to as *ambient PM exposure* at the centroid of census tract of residence of a subject.

Second, numerous studies show that PM exposure is associated with LBW [3,10,11]. Since fetal exposure to PM is possible through circulatory system and only fine particulate matter (PM_2.5_) reaches alveoli and hence to the circulatory system, it is hypothesized that PM_2.5_ exposure will show a significant association with birthweight not PM_10_. It is further hypothesized that PM_2.5_ exposure early in gestation period (e.g., 1st trimester) will show a stronger association with BW than the exposure during the late stage of gestation period (e.g., 2nd and 3rd trimester), because the fetus size is relatively small during early gestation period, and the same PM_2.5_ exposure during early stage of gestation period will translate to high dose given the smaller fetus size. The remainder of this paper presents a methodology, results of the analyses, and a discussion of the findings of this paper within the relevant literature.

## 2. Materials and Methods

### 2.1. Data

This study relies on data from three sources: (a) PM data from both Illinois (IL) and Ohio (OH) states; (b) data on all live births from the Chicago Department of Public Health; and (c) the 2000 US Census data. PM data monitored at all sites in IL and OH (two adjacent States) [12] were used to assess exposure uncertainty, and PM data from Chicago to quantify PM exposure for BW and LBW analysis. All live births (*N* = 398,120) to mothers, who resided in Chicago MSA (that included Cook, DuPage, Kane, Lake, McHenry, and Will counties), were extracted from the Illinois Department of Public Health annual birth certificate records whose gestation period was fully within 2000 to 2004. Each record included a clinical estimate of gestational age (which was utilized to determine different trimesters: 1–13 weeks, 14–26 weeks and 27 weeks to birth), birth weight, date of birth, gender, street address, census tract (Chicago residents only), zip code, city and county of residence at the time of birth, prenatal care (measured by Kessner’s Adequacy of Prenatal Care Index), maternal age, maternal race/ethnicity, marital status, maternal education and country of origin, maternal alcohol and tobacco use during pregnancy, parity, time interval between pregnancies, maternal weight gain, delivery method, maternal medical risk factors, and congenital anomalies in the newborn. Plural births (3.9%), birth weight less than 500 grams (0.3%), or impossible clinical estimates of gestational age and weight combinations (1.0%) were excluded. Although births were geocoded using street address by the Chicago Department of Public Health, these data were acquired and analyzed by census tract centroid (XY Coordinates) due to confidentiality issues. Of the total birth records, only 0.9% of new births were not geocoded or were outside the census tract range of the study area.

The vital records do not include the socio-demographic characteristics of the neighborhood of residence. However, the literature suggests that the neighborhood environment is an important determinant of pregnancy outcomes [8,13,14]. Therefore, the tract level the 2000 US Census data were used to assess the neighborhood socio-economic and demographic characteristics, including household income, poverty level, medial real estate taxes, % minority population etc. In the final analysis, poverty level, measured by % of households receiving public assistance in the Census Tract, was used as a proxy of neighborhood socio-demographics. Because, in the exploratory analysis the percentage of households receiving public assistance was a representative variable, as it showed a significant association with other socio-economic and demographic variables, and also showed the strongest (among the rest of census variables) association with the risks of BW and LBW.

### 2.2. Methodology

#### 2.2.1. Uncertainty Assessment

Spatiotemporal autocorrelation and semivariance were used to explore and quantify exposure uncertainty. Autocorrelation represents the strength of association within the selected distance and time interval, and it captures the degree of homogeneity. A perfect correlation or very high correlation (e.g., >0.95) indicates that there is little variability in PM within the chosen distance and time interval, and PM exposure must be same within this selected spatiotemporal interval (or domain) around a monitoring site. Unlike autocorrelation, semivariance, which is the squared difference of two data points located within a given distance and time interval, captures spatiotemporal heterogeneity. In-situ (or monitored) daily PM_10_ and PM_2.5_ data at all sites in Illinois and Cleveland, OH from 2000 to 2014, were used to compute exposure uncertainty at a given distance (*s*) and time (*l*) interval. Spatiotemporal semivariance (${\hat{\gamma }}_{\left(s,l\right)}$) and autocorrelation ($\rho_{\left(s,l\right)}$) at different distance (*s*) and time (*l*) intervals were computed using a customized program written in C++. Let *A_k,t_* denotes PM observed over D × (t, …, T) where D⊂R^2^ denotes the spatial domain (Illinois and Cleveland) and *t* indexes discrete time stamps (days). If distance threshold is partitioned into *s* intervals, for *s* = (0, 1, 2, …, S), and time lag is partitioned into *l*, for *l* = (0, 1, 2, …, *L*), spatiotemporal autocorrelation (*ρ_(s,l)_*) for a given spatiotemporal interval can computed as the ratio of the covariance (*A_kt_,A_j,t±l_*) to variance of *σ^2^_A_*:

(1) $$\rho_{\left(s,l\right)}=\frac{1}{\sigma_{A}^{2}}\frac{\sum_{k\in D,t\in T}\sum_{j\in D,t\in T}\forall_{\left(k,j\right)\left(t\pm l\right)}\left(A_{k,t}-\hat{A}\right)\left(A_{j,t\pm l}-\hat{A}\right)}{\sum_{k\in D,t\in T}\sum_{j\in D,t\in T}\forall_{\left(k,j\right)\left(t\pm l\right)}}$$

where $\forall_{\left(k,j\right)\left(t\pm l\right)}\;=1$ if the distance between *k^th^* point and its *j^th^* neighbor and time interval between *t^th^* and *l^th^* time lag is ≥∆_(*s−1,l−1*)_ and <∆_(*s,l*)_, 0 otherwise. $\hat{A}$ and $\sigma_{A}^{2}$ are mean and variance of air pollution (either PM_2.5_ of PM_10_ for this study), respectively. Semivariance (*γ_kj,t±l_*) of *k^th^* point on *t^th^* day with respect to *j^th^* neighbor at *l^th^* time interval is squared difference of their values, as *γ* ~ (*A_kt_* − *A_j,t±l_*)^2^. Extending this formula further, average semivariance ($\hat{\gamma }$*_sl_*) within a given distance (*s*) and time interval (*l*) can be computed as:

(2) $${\hat{\gamma }}_{\left(s,l\right)}=\frac{1}{2}\frac{\sum_{k\in D,t\in T}\sum_{j\in D,t\pm l}\forall_{\left(k,j\right)\left(t\pm l\right)}{\left(A_{k,t}-A_{j,t\pm l}\right)}^{2}}{\sum_{k\in D,t\in T}\sum_{j\in T,t\pm \in T}\forall_{\left(k,j\right)\left(t\pm l\right)}}$$

Since each data point is computed twice, numerator is divided by 2 to compute semivariance.

From Equation (2), *ρ_(s,l)_* shows the degree of similarity between data point *A_k,t_* and adjacent neighboring data points within *l^th^* time lag and *s^th^* geographic distance interval, which is simply the ratio of COV(*A_kt_, A_j,t±l_*) to *σ*^2^*_A_*; where COV is covariance. If *A_k,t_* and *A_j,t±l_* are same, *ρ_(s,l)_* ~ 1. Further, *ρ_(s,l)_* can be rewritten as, *ρ^2^_(s,l)_* = 1 − UEV; where UEV is unexplained variance. Since UEV is a measure of uncertainty (*U*) and we can compute *ρ*^2^*_(s,l)_* using the in-situ monitored data, we can quantify *U_(s,l)_* at a given distance and time interval as:

U_(*s,l*)_ ~ 1 − *ρ*^2^_(*s,l*)_ (3)

#### 2.2.2. Exposure Assessment

**Figure 1:**
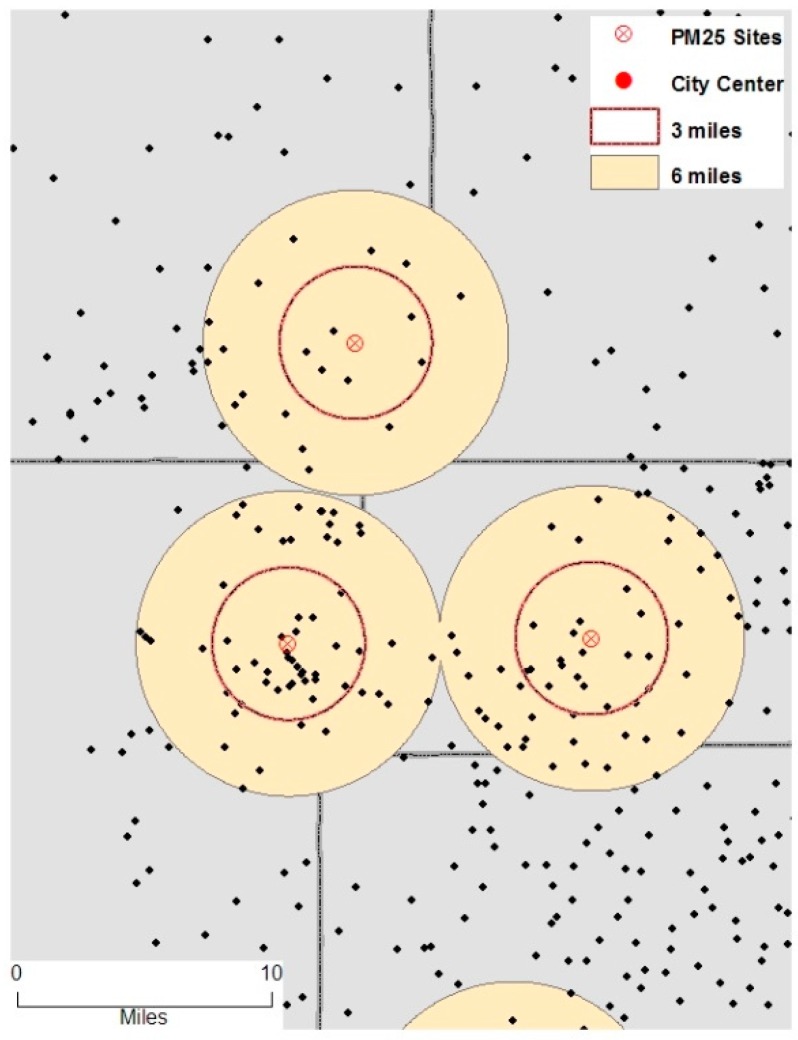
An example of PM_2.5_ monitoring sites and inclusion of subjects within 3 and 6 mile distance radius.

The geocoded births were census track centroid point locations *i* = 1, …, *N*. There were several monitoring stations in some Census Tracts and none within 3 mile or 6 mile distance from the centroid of many Census Tracts (Figure 1). Exposure was computed using only 3 and 6 miles interval between mother’s residence tract and PM monitoring sites. The time interval for exposure computation remained constant for different trimesters and entire pregnancy for each mother. Let *A_kt_* denote PM monitored on days *t* = (1, …, *T*) at spatially dispersed sites *k* = (1, …, *K*) then the daily average PM exposure of mothers (*A_im_*_(*t−L*)_) at location *i* = (1, …, *N*) who gave births on *t^th^* day and lived in census tract *m* = (1, …, *m*) during the gestational length (*L*, measured in days) can be calculated as:

(4) $$A_{im\left(t-L\right)}=\frac{1}{\sum_{l\in L}\sum_{k\in K}\forall_{jk}}\sum_{l\in L}\sum_{k\in K}A_{k\left(t-l\right)}\forall_{jk}$$

where *l* = days before the birth date (*t*); $\forall_{jk}$ = 1 if the distance between *j^th^* census tract centroid and *k^th^* monitoring site was ≤ *s*, 0 otherwise.

#### 2.2.3. Statistical Analysis

Linear and logistic regressions were used to examine BW (measured in gram (g)) and low birth weight (LBW) (coded as a binary variable, 0 = normal, 1 = BW < 2500 g) with respect to PM exposure without and with confounders for each trimester and for entire pregnancy separately. BW (*y_im_*) of a child to *i^th^* mother in *m^th^* census tract, can be modeled as:

*y_im_* ~ *α* + *βA*_*im*|*t−L*|_ + *ε_im_* (5)

where *β* is the coefficient of average PM exposure during gestation period (*L*) and *ε_im_* is random error. Many factors other than PM exposure can affect both BW and LBW, including socio-economic and demographic characteristics of subjects and the place where they live and work during pregnancy. While it was not feasible to acquire characteristics of the work place in the absence of work address (as these data were not available in the vital-record database), socio-demographic characteristics around the place of residence were acquired from the 2000 US Census based on the residential tract of the subjects. The Equation (5) was extended to adjust for subjects-specific socio-economic and demographic characteristics (**C**), such as age, education, smoking and alcohol consumption during pregnancy, and track-specific socio-economic characteristics (**N**), e.g., neighborhood level poverty, as:

*y_im_* ~ *α* + *βA*_*im*|*t−L*|_ + **θ′C′**_*im*_ + **Ψ′N**′_*m*_ + (*υ_m_* + *ε_im_*) (6)

where **θ′** is the vector of coefficients of subject specific socio-demographic characteristics; **Ψ′** is a vector of coefficients of the census tract-specific socio-economic characteristics. *υ_m_* is tract-specific clustering effect that account for intra-tract autocorrelation in exposure and socio-economic conditions. It was important to control for census-track specific clustering, because subjects living in a given tract are likely to have similarities in their socio-economic conditions, which can result in spatial autocorrelation. Moreover, all subjects who resided in the same census tract were assigned the same values of census-track level variables. Since types and sources of PM are likely to be homogeneous within a census tract, there should be greater similarities in PM exposure of subjects within a census tract as well. Logistic regression was used to model LBW in which LBW was coded as 1 if BW < 2500 g, 0 otherwise. All models were run separately for exposure during each of the three trimesters and for the entire gestation controlling for confounders and neighborhood effects, but excluding for exposure uncertainty, addressed below. Both linear and logistic models were implemented in STATA (StataCorp LP, College Station, TX, USA) [15]. In all analyses, only full-term births (≥37 weeks of gestations), because inclusion of pre-term birth which are likely to be low weight can confound the effect of PM exposure.

#### 2.2.4. Accounting for Exposure Uncertainty

If uncertainty is known (as from Equation (3)), the classical Berkson’s measurement error model can be used to account for uncertainty [16]. Since exposure is not the true measurement, rather an estimate at a given location and time, estimated exposure (*A_im,t−L_*) from Equation (4) can be rewritten with the true exposure (X*_im,t−L_*) and the measurement error (U), as:

*A_im,t−L_* ~ X_*im,t−L*_ + U_*im,t−L*_ (7)

Further, substituting exposure with the measurement error, the Equation (6) can be rewritten as:

*y_im_* ~ *α + β*(X_*im,t−L*_ + U_*i,t−L*_) + **θ′C′**_*im*_ + **Ψ′N**′_*m*_ + (*υ_m_* + *ε_im_*) (8)

Rearranging the Equation (8), the final model has three error components as:

*y_im_* ~ *α + β* X_*im,t−L*_ + **θ′C′**_*im*_ + **Ψ′N′**_*m*_ + (*β* U_*i,t−L*_*+ υ_m_* + *ε_im_*) (9)

As shown in Equation (9) there are three error components, including the measurement error. The final model (9) that accounted for measurement errors, confounders and neighborhood effects was implemented in STATA [15] using the SIMEX package [17], which allows the use of exposure with the known level of uncertainty. Based on the empirical estimates, 38.5% and 50% uncertainty was added in the PM_2.5_ and PM_10_, respectively, using a Gaussian distribution, and BW and LWB were modelled with respect to PM exposure with the known uncertainty.

## 3. Results

### 3.1. Exposure Uncertainty Analysis

**Figure 2:**
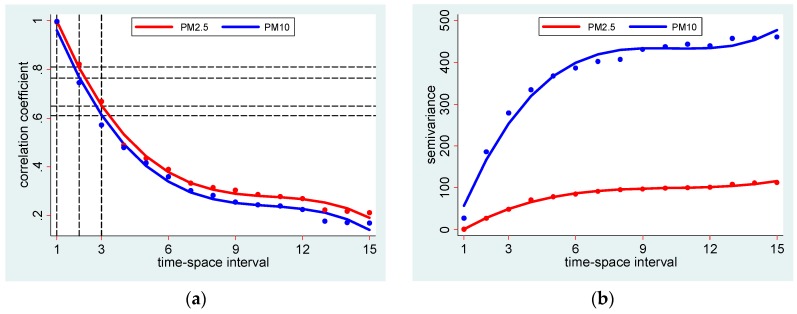
(**a**) Spatiotemporal autocorrelation and (**b**) semivariance of daily PM_2.5_ and PM_10_ in IL and Cleveland, OH. Time-space interval refers to diagonal interval; 1 = time interval ≤1 day and distance interval ≤0.025°, 2 = time interval ≤2 days and distance interval ≤0.05°, …, 15 = time interval ≤15 days and distance interval ≤0.375°.

Exposure uncertainty was examined with the aid of spatiotemporal autocorrelation and semivariance. Using the daily PM data of IL and OH from 2000 to 2014 autocorrelation and semivariance were computed at different distance and time intervals. In IL, there were 38 and 22 sites where PM_2.5_ and PM_10_ were monitored respectively, and in OH 39 and 44 sites, respectively. There more than 40,000 data points in each of these two states for each PM type, suggesting sufficiently large dataset to assess spatiotemporal analysis. The analysis of these data suggests that there is a sharp decline in spatiotemporal autocorrelation and steep rise in semivariance with respect to increase in distance and time intervals (Figure 2a,b; Table 1; see Supplementary Materials Tables S1 and S2, and Figure S1 for details). For example, autocorrelation of PM_2.5_ and PM_10_ in Illinois within 1 day and 0.025° distance is 0.99 and 1.0 respectively, which drops to 0.89 and 0.75 within 2 days and 0.05°. The regression analysis shows that the strength of autocorrelation declines by 0.258 and 0.26 with a unit increase in distance and time interval.

As shown in Table 1, there is a significant decline in the autocorrelation and increase in semivariance (see Tables S1 and S2) with the increase in distance and time intervals. For example, regression coefficient of autocorrelation declines by 0.208 and 0.543 with a unit increase in distance and time intervals (i.e., 0.025° distance and 1 day) for PM_2.5_ and PM_10_, respectively, and the regression coefficient of PM_10_ is more than two times higher than that of PM_2.5_. However, temporal heterogeneity is significantly lower for both PM_2.5_ than for PM_10_. Moreover, time-space interaction term also shows a significant decline for autocorrelation and semivariance of PM_10_, but not for PM_2.5_, further suggesting greater spatiotemporal heterogeneity in PM_10_ as compared to PM_2.5_ (Figure S1 in SOM). Moreover, a comparison of regression coefficients suggests that PM_10_ is >2.5 times more heterogeneous geographically than PM_2.5_ (Table 2). However, PM_2.5_ shows greater temporal heterogeneity than PM_10_, which requires further investigation to understand temporal heterogeneity in PM_2.5_.

As shown in Figure 2a, polynomial fitted autocorrelation at spatiotemporal interval 2 (i.e., 2 days and 0.05° distance) was 0.81 (or R^2^ ~ 0.66) for PM_2.5_ and 0.76 (R^2^ ~ 0.58) for PM_10_, respectively. As in Equation (4), autocorrelation is the ratio of covariance to variance, autocorrelation (*ρ*^2^_(*s,l*)_) ~ R^2^. This means the covariance of PM_2.5_ and PM_10_ within 2 day time interval and 0.05° distance accounts for only 66% and 58% of the total variance of PM_2.5_ and PM_10_, or 34% and 42% unexplained variance (i.e., uncertainty) if these thresholds are used to compute exposure.

### 3.2. Exposure Uncertainty Assessment

Using the results from Table 1, uncertainty level for different thresholds were estimated (Table 3). Computing exposure at a distance of 0.058° (from the monitoring site) will have 38.5% uncertainty in PM_2.5_ exposure and 50% uncertainty for PM_10_. Due to propagation of uncertainty with the increase in distance and time interval, the final analysis was restricted to 0.058° for PM_10_ and 0.116° for PM_2.5_, respectively, and subjects outside these intervals were excluded from the analysis.

### 3.3. Descriptive Analysis of BW, LBW and PM Exposure

**Figure 3:**
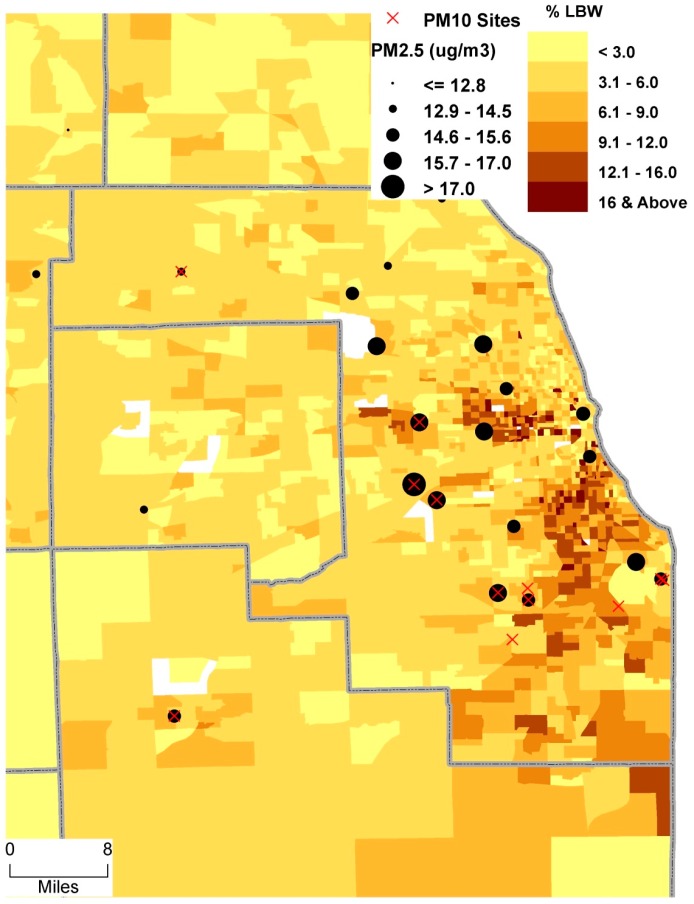
Prevalence of low birth weight (<2500 g) in Chicago MSA, 2000–2004.

This study focuses on Chicago MSA (Figure 3), which is diverse in terms of spatiotemporal distribution of PM, and socio-economic and demographic characteristics. According to the 2000 US Census, about 20% of the population in the study area was white, 39% Hispanic, and 20% African American. The average daily PM_2.5_ exposure during the entire gestation (18 µg/m^3^) was significantly greater than the current annual EPA standards of 12 µg/m^3^ [18]. However, the value for PM_10_ was well within the EPA standards.

The average birth weight in Chicago MSA was 3344.8 g (3343.1 g to 3346.5 g at 95% confidence interval (CI); see Table S3 for details). The value for the city of Chicago was 113 g less than that for the surrounding counties. The analysis further suggests that the odds of LBW for the City of Chicago was 1.59 times higher than the surrounding counties. Overall, the results of descriptive analysis, except PM exposure, are in agreement with the previous literature, for example BW and LBW vary significantly by marital status, smoking, maternal age at birth and educational level (see Kumar [8] and Table S4 for details). The prevalence of LBW in the study area was 5.8%, well below the national rate of 7.0%. However, the incidence of LBW varies within the study areas, ranging from <6% in sub-urban areas to more than 9% in southern east and northern west part of the city (Figure 3). More detailed results of BW and LBW with respect to socio-demographic characteristics are available elsewhere [8].

### 3.4. Effect of PM on BW and LBW

BW (as a continuous variable) and LBW (coded as 0 = normal weight, and 1 low birthweight) were modelled using linear and logistics regressions, respectively. All models were adjusted for some confounders, e.g., age, marital status, education, neighborhood level poverty and smoking status. Among the autocorrelated variables, the variable that showed the strongest association with BW was included, e.g., both smoking and alcohol consumptions showed inverse and strong association with BW. But only smoking that showed stronger association than alcohol was included in the final model to avoid autocollinearity among confounders. Both sets of analyses were run separately without (as in Equation (6)) and with the exposure uncertainty (as in Equation (9)) in order to demonstrate the effect of exposure uncertainty in BW and LBW risk assessment. The results of analyses are presented in Table 4 and Table 5. Since the focus of the paper is on BW and LBW and per-term birth (PTB) are likely to have low birth weight, all analyses were restricted to full-term births (i.e., ≥37 weeks of gestation) to avoid the influence of confounding due to PTB. The effects of air pollution exposure on PTB are available elsewhere [8].

Among the confounders marital status, age, smoking and neighborhood level poverty were significantly associated with BW (Table 4). All these variables except neighborhood poverty were significantly associated with LBW (Table 5). For example, odds of LBW for unmarried women was 68% higher than for married women, and the odds of LBW for smoker was 2.7 time higher than for non-smokers (Table 5). Likewise, BW for smokers and unmarried women was 172 g and 75 g less than for non-smokers and married women, respectively.

As evident from Table 4, PM_2.5_ did not show a significant association with BW at 5% or lower level of significance in the analysis not adjusted for exposure uncertainty. However, in the adjusted for exposure uncertainty analysis PM_2.5_ exposure showed a significant inverse association with BW (i.e., 1 µg/m^3^ PM_2.5_ increase was associated with 0.97 g decrease in birthweight during the entire gestation period). Although the effect of PM_2.5_ exposure during the entire gestation period was highest, among three trimesters the regression coefficient for the exposure during the first trimester was the most significant, which confirms our hypothesis that PM_2.5_ exposure during the early stage of gestation period is stronger predictor of BW than the exposure during the later stages of gestation period. The exposure during the third trimester was not even significant at 5% levels of significance. In the categorical analysis of LBW, neither PM_2.5_ nor PM_10_ showed a significant association with LBW.

## 4. Discussion

Earlier research showed that the risk of BW and LBW associated with PM_10_ exposure changed as distance of mother’s residence to air pollution monitoring station(s) changed [8]. Other studies have found similar results concerning inconsistency, for example change in the risk of PTB with respect to greenness computed at different distance intervals [19]. While such a biological plausibility does not exist because the risk of BW and LBW are likely to remain the same within the same population irrespective of distance to the monitoring stations or distance buffers, this change in risk of LBW with respect to air pollution is attributed to exposure uncertainty [8]. This paper presents a methodology to assess exposure uncertainty at given distance and time interval using in-situ monitored data, which makes two important contributions to the literature. First, using the magnitude of uncertainty, researchers can choose appropriate distance and time-interval to compute exposure that will minimize uncertainty in health risk estimation. For example, the exposure estimation in the present study was restricted to subjects within 0.116° distance interval of monitoring sites, which entailed ~45% uncertainty in PM_2.5_ exposure. Second, once the exposure uncertainty is known, researchers can account for it to reliably estimate the health risk associated with the exposure. Since exposure uncertainty can serve as a proxy of “measurement-error”, it can be implemented using Berkson’s measurement error [16]. However, accounting for the measurement error requires prior knowledge of the error distribution. Generally, measurement error is assumed to be a Gaussian distribution. This may not be true because exposure uncertainty gradually declines with the increase in distance and time intervals. Therefore, it is important to use time-space decay function (or the distribution evident from the distribution of autocorrelation with respect to time and space intervals) to simulate uncertainty if time and space intervals are continuous. However, a Gaussian distribution useful if exact locations of monitoring sites and health data sets are unknown.

With regard to the known socio-economic and demographic covariates, the risks of BW and LBW reported in this paper are consistent with the previous studies [13,20]. However, the findings of the association between BW and PM exposure adjusting for exposure uncertainty and confounders make an important contribution because, this paper shows that the model adjusted for exposure uncertainty, PM_2.5_ exposure showed a significant inverse association with BW, which is consistent with some studies and inconsistent with others. For example, Stieb et al. [11] showed a decline of 20.5 g birth weight with 10 µg/m^3^ increase in PM_2.5_ during the gestation period. The present study shows about 10g decline in birth weight with 10 µg/m^3^ increase in PM_2.5_ during the gestation period. Given Canadians spent more time indoors than Americans, Stieb et al. seem to overestimate the risk of BW associated with outdoor PM_2.5_ exposure. Moreover, they do not account for exposure measurement error. However, the findings of the present study are quite the opposite of those reported by Parker et al. [5], showing a 1.3 g decline in BW with a unit increase in PM_10_ (at county level), and insignificant association between PM_2.5_ exposure and BW.

Although there is a greater interest in quantifying the risk of adverse health outcomes associated with ambient air pollution and many studies have been conducted during the last decade [5,21,22,23], our understanding of the effects of air pollution on birth outcomes (other health effects) is still inconsistent [3]. A vast majority of studies conducted in recent years show that research design and selection bias (i.e., restricting the cases included in the analysis to certain areas and groups) can greatly over- or under-estimate the association between air pollution and birth outcomes. However, most of these studies fail to account for exposure uncertainty. Generally, ad-hoc approaches to select spatial-temporal intervals or buffer zones (such as 3 miles or 30 miles) [24] or indirect (or proxy) measures (such as distance to closest road) [25] are especially responsible for uncertainty in exposure estimation. This paper shows that spatiotemporal autocorrelation and semivariance analyses can be conducted to explore and assess the magnitude of uncertainty. Using this approach, distance and time intervals can be chosen depending on the margin of uncertainty a research can accept. Moreover, this knowledge about uncertainty (in exposure) can be accounted for in assessing the health risks of exposure, as demonstrated in this paper. For example, in the unadjusted model, PM_2.5_ did not show a significant association with BW, but this association became significant when adjusted for this uncertainty.

While this paper advances the literature on air pollution exposure uncertainty and provides insight into the association of PM exposure with BW and LBW, the findings of the papers must be interpreted in the light of below limitations. First, the focus of this study on Chicago and Cleveland limits the scope of generalizability. However, the methodology presented to quantify exposure uncertainty and how to control for it can be used for analyzing data from any other region. Second, the association of PM_2.5_ and PM_10_ exposure with BW and LBW was examined separately, because PM_2.5_ and PM_10_ monitors were not collocated and it was not possible to compute PM_2.5_ and PM_10_ for the same sample and sub-samples in different distance and time intervals. Moreover, exposure uncertainty for PM_10_ and PM_2.5_ was not the same. Therefore, the findings cannot be extrapolated to the synergistic effects of both PM_2.5_ and PM_10_. Moreover, PM exposure does not exist in isolation. Generally, we are exposed to a mixture of particulate and gaseous pollutants, which are not included in the analysis. Third, the focus of this research was on ambient PM, which represents less than 10% of the personal exposure, especially in temperate climate regions like Cleveland and Chicago. Fourth, the effects of PM exposure were not fully adjusted for gestation age despite the fact that PTB were excluded from the analysis, because gestation age is shown to affect birth weight even for the full-term births. Fifth, the analyses of BW and LBW were restricted to a sub-sample because of sparse data. This restricts the scope of generalizability of these findings to the entire Chicago Metropolitan areas. In addition, the results were adjusted for only limited number of confounders, such as age, smoking, and alcohol consumption.

This paper calls for a revised approach to environmental epidemiological studies if the spatiotemporal scales of environment and health data are different and if these data sets do not align spatially and temporally. It is important to understand exposure uncertainty prior to the selection of distance and time interval (or buffer) threshold for computing exposure, and account for this uncertainty in estimating health risks of exposure because failing to account for exposure uncertainty can result in false positive and false negative health risk of environment [8,26]. However, assessment of exposure uncertainty largely depends on the availability of in-situ monitored data. If such data are not available, indirect methods such as satellite data which has daily global spatial coverage can be used to estimate air quality [27].

## 5. Conclusions

Autocorrelation and semivariance analyses suggest that spatial heterogeneity in PM is greater than the temporal heterogeneity. As hypothesized, spatial heterogeneity in PM_10_ is significantly higher than that in PM_2.5_. The gradient of both PM_2.5_ and PM_10_ is steep with the increase in distance and time intervals. Even within very narrow distance, i.e., 0.025° (or about 2.5 km) and ≤1 day time interval, there is about 10% uncertainty in PM exposure. Therefore, distance and time intervals around the monitoring sites must be chosen with caution to compute PM exposure.

In the adjusted model (controlling for confounders and exposure uncertainty) a unit increase in PM_2.5_ exposure was associated with 0.97 g decline in birth weight (or about 9.7 g decline with respect to 10 µg/m^3^ increase in PM_2.5_). Moreover, exposure during the early phase of pregnancy showed a stronger association with BW. As hypothesized, PM_10_ exposure did not show any significant association with either BW or LBW. Other known confounders, such as smoking and marital status, were found to be important risk factors of BW and LBW, which further substantiates the literature on the role of these risk factors on BW and LBW.

## Figures and Tables

**Table 1:** Regression of autocorrelation and semivariance with respect to distance and time intervals.

Variables	Autocorrelation		Semivariance	
	PM_2.5_	PM_10_	PM_2.5_	PM_10_
Distance (°)	−0.208 ***	−0.543 ***	40.65 ***	207.33 ***
Time (day)	−0.043 ***	−0.036 ***	5.71 ***	20.63 ***
D × T	0.005	0.017 ***	−0.77	−9.93 **
Constant	0.796 ***	0.735 ***	29.29 ***	184.18 ***
Observations	600	600	600	600
R^2^	0.709	0.717	0.65	0.64

*** *p* < 0.01, ** *p* < 0.05; D × T = Interaction of Distance and Time.

**Table 2:** Polynomial trend of autocorrelation with respect time-space intervals.

Distance and Time Lag	PM_2.5_	PM_10_
Time-space interval (value ranges between 1 and 15, and increment by 1) ^1^	−0.258 ***	−0.260 ***
	(−0.289–−0.226)	(−0.299–−0.222)
Time-space interval × Time-space interval	0.024 ***	0.024 ***
	(0.019–0.028)	(0.019–0.030)
Time-space interval × Time-space interval × Time-space interval	−0.001 ***	−0.001 ***
	(−0.001–−0.001)	(−0.001–−0.001)
Constant	1.233 ***	1.198 ***
	(1.173–1.294)	(1.125–1.271)
Observations	15	15
R^2^	0.994	0.992

*** *p* < 0.01, (95% confidence interval in parenthesis). ^1^ one increment of time-space lag ~0.025° distance and one day time interval.

**Table 3:** Exposure uncertainty using the regression of PM autocorrelation with respect to different spatiotemporal interval.

Distance	PM_2.5_ (α ~ 0.796; β ~ −0.232)			PM_10_ (α ~ 735; β ~ −0.553)		
	ρ *	ρ^2^	% Uncertainty **	ρ	ρ^2^	% Uncertainty
0.058°	0.784	0.61	38.5	0.703	0.494	50.6
0.116°	0.772	0.60	40.4	0.671	0.450	55.0
0.174°	0.760	0.58	42.3	0.639	0.408	59.2
0.232°	0.748	0.56	44.1	0.607	0.368	63.2

* ρ ~ *α* + *βd_s_*; ** % uncertainty (*U*) ~ 100 × (*1 − ρ*^2^).

**Table 4:** Linear regression of BW (g) with respect PM exposure in Chicago, 2000–2004 without and with the exposure measurement error.

Variables	Naïve (Linear Regression with Neighborhood Specific Effect Equation (6))				SIMEX Model (Accounting for Exposure Uncertainty, Equation (9))			
	1–2 ^a^	2–3	3–4	1–4	1–2	2–3	3–4	1–4
**PM_2.5_ (µg/m^3^)**								
Marital Status (0 = married, 1 otherwise)	−65.54 ***	−65.14 ***	−65.07 ***	−65.25 ***	−65.54 ***	−65.15 ***	−65.07 ***	−65.26 ***
	(−75.44–−55.64)	(−75.10–−55.17)	(−75.05–−55.09)	(−75.22–−55.28)	(−73.75–−57.34)	(−73.19–−57.11)	(−73.57–−56.57)	(−73.78–−56.74)
Age Groups (1–4; coded in ascending order)	47.92 ***	48.45 ***	48.31 ***	48.46 ***	47.92 ***	48.44 ***	48.31 ***	48.45 ***
	(42.24–53.60)	(42.81–54.08)	(42.72–53.90)	(42.85–54.06)	(42.60–53.24)	(43.23–53.66)	(43.75–52.86)	(43.03–53.87)
Mother’s Education (coded in ascending order)	3.795	3.496	3.456	3.5	3.786 *	3.479 *	3.453 *	3.490 **
	(−1.050–8.641)	(−1.355–8.346)	(−1.389–8.301)	(−1.328–8.327)	(−0.0898–7.661)	(−0.186–7.145)	(−0.299–7.204)	(0.354–6.625)
ln (% households receiving public assistance in the census tract of mother’s residence)	−18.27 ***	−17.50 ***	−17.25 ***	−17.24 ***	−18.27 ***	−17.49 ***	−17.25 ***	−17.23 ***
	(−24.24–−12.30)	(−23.52–−11.48)	(−23.29–−11.20)	(−23.26–−11.21)	(−21.78–−14.76)	(−21.32–−13.66)	(−21.04–−13.46)	(−21.10–−13.37)
Smoking (0 = no, 1 = yes)	−172.5 ***	−173.1 ***	−172.4 ***	−172.5 ***	−172.5 ***	−173.1 ***	−172.4 ***	−172.5 ***
	(−190.4–−154.6)	(−190.9–−155.3)	(−190.2–−154.6)	(−190.3–−154.7)	(−189.2–−155.7)	(−189.1–−157.0)	(−187.6–−157.1)	(−186.1–−158.9)
PM_2.5_ exposure (µg/m^3^)	−0.984 *	−0.851 *	−0.685	−1.007 *	−0.955 **	−0.799 **	−0.676 *	−0.976 ***
	(−1.994–0.0253)	(−1.815–0.113)	(−1.570–0.199)	(−2.017–0.00310)	(−1.779–−0.131)	(−1.520–−0.0777)	(−1.445–0.0938)	(−1.689–−0.263)
Observations	60,774	61,272	61,749	61,929	60,774	61,272	61,749	61,929
R^2^	0.035	0.035	0.034	0.035				
**PM_10_ (µg/m^3^)**								
Marital Status (0 = married, 1 otherwise)	−67.37 ***	−65.32 ***	−65.26 ***	−67.32 ***	−67.37 ***	−65.33 ***	−65.26 ***	−67.32 ***
	(−98.19–−36.55)	(−97.13–−33.51)	(−96.73–−33.80)	(−97.95–−36.69)	(−94.93–−39.80)	(−84.68–−45.98)	(−90.07–−40.45)	(−94.84–−39.80)
Age Groups (1–4; coded in ascending order)	61.11 ***	62.26 ***	62.63 ***	61.17 ***	61.11 ***	62.26 ***	62.64 ***	61.16 ***
	(44.08–78.14)	(45.21–79.32)	(45.44–79.82)	(44.09–78.25)	(45.14–77.08)	(47.46–77.06)	(46.67–78.62)	(45.18–77.14)
Mother’s Education (coded in ascending order)	−2.154	−1.613	−1.777	−2.167	−2.163	−1.613	−1.78	−2.17
	(−17.51–13.20)	(−17.11–13.88)	(−17.24–13.68)	(−17.50–13.17)	(−12.26–7.935)	(−12.55–9.322)	(−10.56–7.004)	(−12.27–7.934)
ln (% households receiving public assistance in the census tract of mother’s residence)	−40.58 ***	−42.06 ***	−41.19 ***	−40.24 ***	−40.56 ***	−42.11 ***	−41.13 ***	−40.24 ***
	(−58.38–−22.77)	(−60.02–−24.10)	(−59.61–−22.77)	(−58.91–−21.57)	(−51.83–−29.29)	(−51.84–−32.39)	(−51.02–−31.24)	(−51.43–−29.05)
Smoking (0 = no, 1 = yes)	−176.2 ***	−175.4 ***	−176.6 ***	−176.6 ***	−176.2 ***	−175.3 ***	−176.7 ***	−176.6 ***
	(−214.6–−137.8)	(−213.5–−137.3)	(−214.9–−138.3)	(−214.9–−138.3)	(−208.4–−144.0)	(−210.6–−140.1)	(−211.6–−141.8)	(−208.7–−144.5)
PM_10_ exposure (µg/m^3^)	−0.646	0.0474	−1.146	−1.014	−0.665	0.0967	−1.207	−1.012
	(−2.946–1.654)	(−2.420–2.515)	(−3.875–1.582)	(−4.717–2.689)	(−2.317–0.987)	(−1.522–1.715)	(−2.698–0.284)	(−3.001–0.976)
Observations	8344	8249	8161	8344	8344	8249	8161	8344
R^2^	0.049	0.049	0.05	0.049				

*** *p* < 0.01, ** *p* < 0.05, * *p* < 0.1 (Robust 95% confidence interval in parentheses); ^a^ 1–2 = 1st trimester; likewise, 2–3 represents 2nd trimester and 3–4 represents third trimester.

**Table 5:** Logistic regression of LBW (binary variable) with respect to PM exposure in Chicago, 2000–2004 without and with the exposure measurement error.

Variables	Naïve (Logistic Regression with Neighborhood Specific Effect Equation (6))				SIMEX Model (Accounting for Exposure Uncertainty, Equation (9))			
	1–2 ^a^	2–3	3–4	1–4	1–2	2–3	3–4	1–4
**PM_2.5_ (µg/m^3^)**								
Marital Status (0 = married, 1 otherwise)	1.682 ***	1.685 ***	1.668 ***	1.667 ***	1.682 ***	1.685 ***	1.668 ***	1.667 ***
	(1.427–1.983)	(1.430–1.985)	(1.417–1.963)	(1.416–1.962)	(1.468–1.927)	(1.467–1.935)	(1.455–1.913)	(1.456–1.909)
Age Groups (1–4; coded in ascending order)	1.085 *	1.081	1.079	1.077	1.085 *	1.081 *	1.079	1.077 *
	(0.987–1.192)	(0.984–1.188)	(0.983–1.185)	(0.981–1.183)	(0.995–1.182)	(0.988–1.182)	(0.982–1.186)	(0.995–1.165)
Mother’s Education (coded in ascending order)	0.976	0.979	0.977	0.979	0.976	0.979	0.977	0.979
	(0.913–1.044)	(0.916–1.046)	(0.914–1.044)	(0.916–1.046)	(0.916–1.040)	(0.914–1.048)	(0.924–1.033)	(0.914–1.048)
ln (% households receiving public assistance in the census tract of mother’s residence)	1.038	1.04	1.046	1.047	1.038	1.04	1.046	1.047
	(0.961–1.121)	(0.964–1.122)	(0.970–1.127)	(0.971–1.129)	(0.973–1.106)	(0.974–1.110)	(0.978–1.118)	(0.981–1.118)
Smoking (0 = no, 1 = yes)	2.706 ***	2.741 ***	2.753 ***	2.751 ***	2.706 ***	2.740 ***	2.753 ***	2.751 ***
	(2.255–3.246)	(2.288–3.283)	(2.300–3.296)	(2.298–3.294)	(2.246–3.260)	(2.263–3.317)	(2.306–3.286)	(2.347–3.225)
PM_2.5_ exposure (µg/m^3^)	0.998	0.998	0.997	0.998	0.998	0.998	0.997	0.998
	(0.983–1.012)	(0.985–1.011)	(0.985–1.010)	(0.984–1.012)	(0.985–1.011)	(0.986–1.009)	(0.983–1.012)	(0.983–1.014)
Observations	60,774	61,272	61,749	61,929	60,774	61,272	61,749	61,929
**PM_10_ (µg/m^3^)**								
Marital Status (0 = married, 1 otherwise)	1.432	1.37	1.368	1.43	1.432 *	1.370 *	1.368	1.431 *
	(0.930–2.204)	(0.882–2.127)	(0.883–2.120)	(0.931–2.198)	(0.990–2.070)	(0.957–1.962)	(0.923–2.027)	(0.987–2.074)
Age Groups (1–4; coded in ascending order)	0.872	0.859	0.861	0.87	0.872	0.859	0.861	0.87
	(0.678–1.121)	(0.664–1.111)	(0.664–1.116)	(0.676–1.120)	(0.682–1.114)	(0.677–1.090)	(0.688–1.078)	(0.680–1.113)
Mother’s Education (coded in ascending order)	1	0.995	0.999	1.001	1	0.995	0.999	1.001
	(0.849–1.178)	(0.842–1.176)	(0.845–1.182)	(0.850–1.178)	(0.847–1.182)	(0.837–1.183)	(0.863–1.157)	(0.842–1.189)
ln (% households receiving public assistance in the census tract of mother’s residence)	1.1	1.114	1.121	1.093	1.099	1.115	1.121	1.094
	(0.889–1.362)	(0.897–1.384)	(0.908–1.385)	(0.869–1.374)	(0.921–1.312)	(0.940–1.321)	(0.965–1.301)	(0.917–1.306)
Smoking (0 = no, 1 = yes)	2.908 ***	2.899 ***	2.864 ***	2.930 ***	2.909 ***	2.898 ***	2.866 ***	2.927 ***
	(2.114–4.002)	(2.117–3.969)	(2.078–3.948)	(2.160–3.976)	(1.952–4.334)	(1.929–4.352)	(1.933–4.248)	(1.969–4.352)
PM_10_ exposure (µg/m^3^)	1.01	1.017	1.009	1.017	1.011	1.016	1.009	1.016
	(0.980–1.042)	(0.981–1.054)	(0.976–1.044)	(0.966–1.071)	(0.983–1.040)	(0.992–1.041)	(0.986–1.033)	(0.984–1.048)
Observations	8344	8249	8161	8344	8344	8249	8161	8344

*** *p* < 0.01, * *p* < 0.1 (Robust 95% confidence interval in parentheses); ^a^ 1–2 = 1st trimester; likewise, 2–3 represents 2nd trimester and 3–4 represents third trimester.

## Supplementary Materials

The following are available online at www.mdpi.com/1660-4601/13/9/906/s1. Table S1: Spatiotemporal autocorrelation (semivariance in parenthesis) of daily PM in Illinois from 2000 to 2014 at different distance and time intervals. Table S2: Spatiotemporal autocorrelation (semivariance in parenthesis) of daily PM in Cleveland, OH from 2000 to 2014 at different distance and time intervals. Table S3: Birth weight (g) by different categories of the selected covariates ± 95% confidence interval (CI) and number of observations in the line below, and the difference in the birth weight between reference and other categories (*p*-value in parenthesis). Table S4: Odds of LBW with respect to different socio-economic and demographic covariates. Figure S1: Spatiotemporal autocorrelation and semivariance of PM_2.5_: (a) autocorrelation Illinois; (b) autocorrelation Ohio; (c) semivariance, Illinois; (d) semivariance, Ohio.
