# Supplementary material for: The Exposure Uncertainty Analysis: The Association between Birth Weight and Trimester Specific Exposure to Particulate Matter (PM2.5 vs. PM10)
Source: Int J Environ Res Public Health. 2016 Sep 13;13(9):906. doi: 10.3390/ijerph13090906 (PMC5036739; doi:10.3390/ijerph13090906)
Supplement: Supplementary file 1 [file ijerph-13-00906-s001.pdf]

Int. J. Environ. Res. Public Health **2016**, *13*, 906; doi:10.3390/ijerph13090906

Supplementary Materials: The Exposure Uncertainty Analysis: The Association between Birth Weight and Trimester Specific Exposure to Particulate Matter ($PM_{2.5}$ vs. PM_{10})

Naresh Kumar

Table S1. Spatiotemporal autocorrelation (semivariance in parenthesis) of daily PM in Illinois from 2000 to 2014 at different distance and time intervals.

Time Interval (Days)	Distance Interval (Degree)						
	**≤0.025**	**≤0.05**	**≤0.075**	**≤0.1**	**≤0.125**	**≤0.15**	**≤0.175**
	**Particulate Matter ≤ 10 µm in aerodynamic diameter (PM_{10})**						
≤1 *	1.00 (11.6)	1.00 (18.2)	0.95 (30.1)	0.94 (43.5)	0.93 (44.6)	0.91 (47.9)	0.86 (66.1)
≤2	0.77 (187.6)	0.75 (189.7)	0.71 (194.1)	0.71 (202.0)	0.70 (201.6)	0.70 (200.3)	0.66 (210.1)
≤3	0.60 (286.2)	0.59 (287.3)	0.56 (288.7)	0.56 (295.4)	0.56 (294.8)	0.55 (293.1)	0.52 (299.3)
≤4	0.51 (343.0)	0.50 (343.5)	0.47 (343.4)	0.47 (349.4)	0.47 (348.8)	0.47 (347.1)	0.43 (351.1)
≤5	0.44 (380.9)	0.43 (381.1)	0.41 (380.2)	0.41 (385.9)	0.41 (385.4)	0.41 (383.8)	0.38 (386.9)
≤6	0.40 (404.5)	0.40 (404.4)	0.37 (403.1)	0.37 (408.4)	0.37 (407.9)	0.37 (406.6)	0.34 (408.7)
≤7	0.38 (415.9)	0.37 (415.2)	0.34 (413.1)	0.34 (418.4)	0.34 (417.8)	0.34 (415.9)	0.31 (417.8)
	**Particulate Matter ≤ 2.5 µm in aerodynamic diameter ($PM_{2.5}$)**						
≤1	0.99 (1.5)	1.00 (2.3)	1.00 (3.2)	0.98 (5.5)	0.99 (6.3)	0.99 (7.3)	0.99 (8.7)
≤2	0.89 (15.7)	0.89 (16.7)	0.88 (18.7)	0.87 (19.0)	0.87 (20.6)	0.86 (23.0)	0.86 (23.0)
≤3	0.76 (31.4)	0.75 (32.7)	0.74 (35.6)	0.74 (34.5)	0.74 (36.8)	0.72 (40.1)	0.72 (39.1)
≤4	0.51 (59.7)	0.51 (61.3)	0.51 (63.0)	0.49 (63.9)	0.49 (65.7)	0.48 (67.6)	0.47 (68.2)
≤5	0.47 (64.8)	0.47 (66.3)	0.46 (68.2)	0.45 (68.3)	0.45 (70.4)	0.44 (72.6)	0.43 (72.6)
≤6	0.43 (69.2)	0.43 (70.6)	0.42 (72.8)	0.42 (72.3)	0.41 (74.5)	0.40 (77.1)	0.39 (76.6)
≤7	0.34 (78.3)	0.34 (79.8)	0.34 (81.4)	0.33 (81.6)	0.33 (83.3)	0.32 (85.4)	0.31 (85.5)

*: Since the location of PM monitoring site is sparse, the program will be calculate autocorrelation for a data point with itself if other data points are not found within the chosen distance and time interval, e.g., autocorrelation on the same day within 0.05 degree will compute autocorrelation with itself if no other sites are within this distance.

Table S2. Spatiotemporal autocorrelation (semivariance in parenthesis) of daily PM in Cleveland, OH from 2000 to 2014 at different distance and time intervals.

Time Interval (Days)	Distance Interval (Degree)						
	**≤0.025**	**≤0.05**	**≤0.075**	**≤0.1**	**≤0.125**	**≤0.15**	**≤0.175**
	**Particulate Matter ≤ 10 µm in aerodynamic diameter (PM_{10})**						
≤1 *	0.96 (43.4)	0.91 (61.1)	0.90 (61.5)	0.89 (61.7)	0.88 (62.0)	0.84 (72.5)	0.79 (88.9)
≤2	0.77 (183.8)	0.74 (182.5)	0.74 (181.7)	0.74 (181.2)	0.73 (179.1)	0.68 (184.9)	0.65 (201.7)
≤3	0.60 (277.2)	0.59 (270.3)	0.58 (269.0)	0.58 (268.3)	0.58 (265.3)	0.53 (265.9)	0.51 (282.7)
≤4	0.51 (331.0)	0.50 (322.2)	0.49 (320.8)	0.49 (320.0)	0.49 (316.6)	0.45 (313.4)	0.42 (331.3)
≤5	0.44 (365.9)	0.43 (356.4)	0.43 (354.6)	0.43 (353.7)	0.43 (349.9)	0.39 (344.5)	0.37 (363.0)
≤6	0.40 (390.0)	0.39 (379.5)	0.39 (377.8)	0.39 (377.0)	0.39 (373.1)	0.35 (365.7)	0.33 (383.7)
≤7	0.37 (393.6)	0.36 (385.7)	0.35 (383.7)	0.35 (382.4)	0.35 (378.2)	0.32 (371.1)	0.30 (386.3)
	**Particulate Matter ≤ 2.5 µm in aerodynamic diameter ($PM_{2.5}$)**						
≤1	1.00 (0.0)	1.00 (0.0)	1.00 (0.0)	1.00 (0.0)	1.00 (0.0)	1.00 (0.0)	0.99 (0.5)
≤2	0.76 (36.3)	0.76 (36.3)	0.76 (36.3)	0.76 (36.4)	0.76 (36.4)	0.76 (36.4)	0.75 (36.2)
≤3	0.59 (61.3)	0.59 (61.4)	0.59 (61.4)	0.59 (61.5)	0.59 (61.5)	0.59 (61.5)	0.59 (60.9)
≤4	0.49 (77.1)	0.49 (77.1)	0.49 (77.1)	0.49 (77.3)	0.49 (77.3)	0.49 (77.3)	0.49 (76.6)
≤5	0.43 (86.8)	0.43 (86.8)	0.43 (86.8)	0.43 (87.0)	0.43 (87.0)	0.43 (87.1)	0.43 (86.2)
≤6	0.39 (93.3)	0.38 (93.4)	0.38 (93.4)	0.38 (93.6)	0.38 (93.6)	0.38 (93.7)	0.38 (92.7)
≤7	0.36 (97.9)	0.35 (98.0)	0.35 (98.0)	0.35 (98.3)	0.35 (98.3)	0.35 (98.4)	0.35 (97.3)

*: Since the location of PM monitoring site is sparse, the program will be calculate autocorrelation for a data point with itself if other data points are not found within the chosen distance and time interval, e.g., autocorrelation on the same day within 0.05 degree will compute autocorrelation with itself if no other sites are within this distance.

Table S3. Birth weight (g) by different categories of the selected covariates ± 95% confidence interval (CI) and number of observations in the line below, and the difference in the birth weight between reference and other categories (*p*-value in parenthesis).

Variable	Variable Categories	Chicago		Outside of Chicago		Both	
		Mean ± 95% CI	Difference ± 95% CI (*p*-Value)	Mean ± 95% CI	Difference ± 95% CI (*p*-Value)	Mean ± 95% CI	Difference ± 95% CI (*p*-Value)
Low Birth−weight	No	3370.2 ± 2.25<br>149,522		3454.2 ± 1.85<br>225,495		3420.7 ± 1.44<br>375,017	
	Yes	2108.8 ± 6.69<br>11,868	1261.4 ± 8.20<br>(0.0000)	2118.0 ± 6.79<br>11,235	1336.1 ± 8.44<br>(0.0000)	2113.3 ± 4.77<br>23,103	1307.4 ± 5.91<br>(0.0000)
Pre−term Delivery	No	3358.3 ± 2.40<br>147,089		3453.0 ± 1.94<br>220,842		3415.1 ± 1.52<br>367,931	
	Yes	2446.2 ± 9.85<br>14,301	912.0 ± 8.30<br>(0.0000)	2526.0 ± 9.41<br>15,888	927.0 ± 7.65<br>(0.0000)	2488.2 ± 6.82<br>30,189	926.9 ± 5.64<br>(0.0000)
Child's Gender	Male	3330.9±3.82<br>82,461		3445.7 ± 3.04<br>121,323		3399.2 ± 2.39<br>203,784	
	Female	3221.7 ± 3.71<br>78,929	109.2 ± 5.33<br>(0.0000)	3333.0 ± 2.94<br>115,407	112.7 ± 4.24<br>(0.0000)	3287.8 ± 2.32<br>194,336	111.4 ± 3.34<br>(0.0000)
Year of Birth	2000	3248.1 ± 11.11<br>10,591		3366.4 ± 9.02<br>14,468		3316.4 ± 7.05<br>25,059	
	2001	3281.6 ± 5.34<br>40,259	−33.5 ± 11.87<br>(0.0000)	3401.1 ± 4.28<br>58,976	−34.8 ± 9.73<br>(0.0000)	3352.6 ± 3.36<br>99,235	−36.2 ± 7.57<br>(0.0000)
	2002	3283.2 ± 5.48<br>38,133	−35.1 ± 11.93<br>(0.0000)	3396.9 ± 4.36<br>56,009	−30.5 ± 9.72<br>(0.0000)	3350.8 ± 3.43<br>94,142	−34.4 ± 7.58<br>(0.0000)
	2003	3275.7 ± 5.56<br>37,183	−27.6 ± 11.99<br>(0.0000)	3388.4 ± 4.40<br>54,867	−22.1 ± 9.73<br>(0.0000)	3342.9 ± 3.47<br>92,050	−26.5 ± 7.60<br>(0.0000)
	2004	3277.1 ± 5.68<br>35,224	−29.0 ± 12.02<br>(0.0000)	3381.6 ± 4.49<br>52,410	−15.3 ± 9.77<br>(0.0022)	3339.6 ± 3.54<br>87,634	−23.2 ± 7.62<br>(0.0000)
Season of Birth	Fall	3272.5 ± 5.15<br>44,087		3389.1 ± 4.09<br>64,194		3341.7 ± 3.22<br>108,281	
	Spring	3285.9 ± 5.55<br>37,689	−13.4 ± 7.57<br>(0.0005)	3400.8 ± 4.34<br>56,767	−11.7 ± 5.96<br>(0.0001)	3355.0 ± 3.44<br>94,456	−13.3 ± 4.71<br>(0.0000)
	Summer	3277.3 ± 5.38<br>39,315	−4.7 ± 7.45<br>(0.2141)	3392.8 ± 4.28<br>58,737	−3.7 ± 5.92<br>(0.2265)	3346.5 ± 3.37<br>98,052	−4.8 ± 4.66<br>(0.0435)
	Winter	3275.1 ± 5.36<br>40,299	−2.6 ± 7.43<br>(0.4998)	3380.4 ± 4.34<br>57,032	8.7 ± 5.96<br>(0.0042)	3336.8 ± 3.39<br>97,331	4.9 ± 4.68<br>(0.0420)
Race/Ethnicity	White	3423.8 ± 5.69<br>32,616		3461.7 ± 2.79<br>133,984		3454.3 ± 2.51<br>166,600	
	Black	3129.7 ± 4.55<br>58,685	294.0 ± 7.43<br>(0.0000)	3182.0 ± 6.82<br>25,286	279.7 ± 7.08<br>(0.0000)	3145.5 ± 3.79<br>83,971	308.8 ± 4.44<br>(0.0000)
	Hispanic	3344.5 ± 4.04<br>63,559	79.2 ± 6.95<br>(0.0000)	3363.8 ± 4.01<br>61,695	97.9 ± 4.93<br>(0.0000)	3354.0 ± 2.84<br>125,254	100.2 ± 3.80<br>(0.0000)
	Other	3221.5 ± 12.06<br>6530	202.3 ± 13.82<br>(0.0000)	3227.9 ± 7.66<br>15,765	233.8 ± 8.56<br>(0.0000)	3226.0 ± 6.47<br>22,295	228.3 ± 7.25<br>(0.0000)
Mother's Age	≤18	3129.1 ± 7.88<br>16,875		3200.1 ± 9.75<br>10,801		3156.8 ± 6.14<br>27,676	
	19–25	3242.2 ± 4.18<br>60,653	−113.1 ± 8.95<br>(0.0000)	3316.4 ± 4.07<br>60,168	−116.3 ± 10.47<br>(0.0000)	3279.2 ± 2.93<br>120,821	−122.4 ± 6.79<br>(0.0000)
	26–34	3333.7 ± 4.25<br>64,680	−204.6 ± 9.24<br>(0.0000)	3426.8 ± 2.91<br>123,173	−226.8 ± 10.24<br>(0.0000)	3394.8 ± 2.41<br>187,853	−237.9 ± 6.71<br>(0.0000)
	≥35	3329.5 ± 8.45<br>19,182	−200.3 ± 11.65<br>(0.0000)	3439.8 ± 5.28<br>42,588	−239.8 ± 11.58<br>(0.0000)	3405.6 ± 4.50<br>61,770	−248.7 ± 7.89<br>(0.0000)
Wight gained during pregnancy	≤25 lbs weight gain	3141.3 ± 5.01<br>50,243		3260.5 ± 4.25<br>65,722		3208.9 ± 3.26<br>115,965	
	26–34 lbs gained	3284.8 ± 4.04<br>63,951	−143.5 ± 6.36<br>(0.0000)	3380.3 ± 3.18<br>96,219	−119.8 ± 5.21<br>(0.0000)	3342.2 ± 2.51<br>160,170	−133.3 ± 4.05<br>(0.0000)
	≥35 lbs gained	3412.5 ± 4.72<br>47,196	−271.1 ± 6.90<br>(0.0000)	3518.5 ± 3.62<br>74,789	−258.0 ± 5.55<br>(0.0000)	3477.5 ± 2.89<br>121,985	−268.6 ± 4.34<br>(0.0000)
Mother's Education	Less than HS	3236.1 ± 4.58<br>54,678		3318.2 ± 4.98<br>41,570		3271.6 ± 3.38<br>96,248	
	High School	3250.2 ± 4.97<br>47,093	−14.1 ± 6.75<br>(0.0000)	3347.9 ± 4.43<br>55,687	−29.7 ± 6.69<br>(0.0000)	3303.1 ± 3.32<br>102,780	−31.6 ± 4.74<br>(0.0000)
	Some college	3278.2 ± 6.54<br>27,711	−42.1 ± 7.94<br>(0.0000)	3384.4 ± 4.73<br>49,328	−66.2 ± 6.89<br>(0.0000)	3346.2 ± 3.85<br>77,039	−74.7 ± 5.12<br>(0.0000)
	College or more	3387.9 ± 5.82<br>31,908	−151.8 ± 7.46<br>(0.0000)	3454.1 ± 3.39<br>90,145	−135.9 ± 6.03<br>(0.0000)	3436.8 ± 2.93<br>122,053	−165.2 ± 4.47<br>(0.0000)
Immigrant	No	3236.3 ± 3.43<br>103,276		3408.1 ± 2.62<br>162,198		3341.3 ± 2.11<br>265,474	

	Yes	3350.6 ± 4.19<br>58,114	−114.3 ± 5.55<br>(0.0000)	3352.9 ± 3.63<br>74,532	55.2 ± 4.58<br>(0.0000)	3351.9 ± 2.75<br>132,646	−10.6 ± 3.56<br>(0.0000)
Married	Yes	3373.4 ± 3.74<br>76,871		3430.9 ± 2.43<br>177,469		3413.5 ± 2.04<br>254,340	
	No	3190.1 ± 3.71<br>84,519	183.3 ± 5.28<br>(0.0000)	3270.5 ± 4.27<br>59,261	160.4 ± 4.87<br>(0.0000)	3223.3 ± 2.81<br>143,780	190.2 ± 3.44<br>(0.0000)
Parity Status	First birth	3244.3 ± 4.29<br>59,019		3319.1 ± 3.65<br>79,924		3287.3 ± 2.79<br>138,943	
	>1 birth	3296.5 ± 3.41<br>102,371	−52.2 ± 5.55<br>(0.0000)	3427.3 ± 2.60<br>156,806	−108.2 ± 4.48<br>(0.0000)	3375.6 ± 2.09<br>259,177	−88.3 ± 3.51<br>(0.0000)
Interval during pregnancy	First pregnancy	3244.3 ± 4.29<br>59,019		3319.1 ± 3.65<br>79,924		3287.3 ± 2.79<br>138,943	
	<1year	3090.9 ± 23.77<br>2962	153.4 ± 19.87<br>(0.0000)	3293.6 ± 18.23<br>4372	25.5 ± 16.19<br>(0.0021)	3211.8 ± 14.67<br>7334	75.6 ± 12.60<br>(0.0000)
	≥1 year	3302.7 ± 3.44<br>99,409	−58.4 ± 5.55<br>(0.0000)	3431.1 ± 2.62<br>152,434	−112.0 ± 4.48<br>(0.0000)	3380.4 ± 2.10<br>251,843	−93.1 ± 3.51<br>(0.0000)
Kessner's Index	Adequate	3312.6 ± 3.21<br>108,746		3412.0 ± 2.36<br>188,720		3375.7 ± 1.91<br>297,466	
	Intermediate	3232.7 ± 5.66<br>36,165	79.8 ± 6.45<br>(0.0000)	3321.5 ± 5.42<br>36,237	90.6 ± 5.89<br>(0.0000)	3277.1 ± 3.93<br>72,402	98.5 ± 4.33<br>(0.0000)
	Inadequate	3143.8 ± 8.84<br>16,479	168.8 ± 8.92<br>(0.0000)	3262.8 ± 10.29<br>11,773	149.2 ± 9.81<br>(0.0000)	3193.4 ± 6.74<br>28,252	182.3 ± 6.54<br>(0.0000)
Smoking	No	3296.4 ± 2.73<br>151,176		3402.1 ± 2.18<br>223,825		3359.5 ± 1.71<br>375,001	
	Yes	2996.6 ± 11.09<br>10,214	299.8 ± 10.89<br>(0.0000)	3193.4 ± 9.25<br>12,905	208.7 ± 9.34<br>(0.0000)	3106.5 ± 7.23<br>23,119	253.1 ± 7.12<br>(0.0000)
Alcohol consumption during pregnancy	No	3279.2 ± 2.68<br>160,579		3391.0 ± 2.13<br>235,722		3345.7 ± 1.68<br>396,301	
	Yes	2930.0 ± 42.61<br>811	349.2 ± 37.81<br>(0.0000)	3337.4 ± 36.60<br>1008	53.5 ± 32.70<br>(0.0013)	3155.8 ± 29.29<br>1819	189.9 ± 24.85<br>(0.0000)
Delivery method	Vaginal	3271.0 ± 2.84<br>127,397		3386.4 ± 2.30<br>179,878		3338.6 ± 1.80<br>307,275	
	Primary c−section	3291.5 ± 9.17<br>21,114	−20.5 ± 7.92<br>(0.0000)	3383.6 ± 7.14<br>32,584	2.8 ± 6.20<br>(0.3687)	3347.4 ± 5.65<br>53,698	−8.8 ± 4.91<br>(0.0005)
	Repeat c−section	3318.1 ± 10.32<br>12,879	−47.0 ± 9.53<br>(0.0000)	3432.3 ± 6.98<br>24,268	−45.9 ± 6.76<br>(0.0000)	3392.7 ± 5.82<br>37,147	−54.1 ± 5.56<br>(0.0000)
At one or more medical risk	No	3299.2 ± 2.98<br>117,892		3409.9 ± 2.37<br>169,797		3364.6 ± 1.87<br>287,689	
	Yes	3218.4 ± 5.73<br>43,498	80.8 ± 6.02<br>(0.0000)	3342.1 ± 4.53<br>66,933	67.9 ± 4.72<br>(0.0000)	3293.3 ± 3.57<br>110,431	71.2 ± 3.74<br>(0.0000)
Congenital anomaly	No	3278.2 ± 2.68<br>159,720		3392.8 ± 2.13<br>233,314		3346.2 ± 1.68<br>393,034	
	Yes	3201.9 ± 31.22<br>1670	76.4 ± 26.45<br>(0.0000)	3250.4 ± 22.21<br>3416	142.4 ± 17.84<br>(0.0000)	3234.5 ± 18.11<br>5086	111.8 ± 14.92<br>(0.0000)

Table S4. Odds of LBW with respect to different socio-economic and demographic covariates.

Variables	# of Births (% in Parenthesis)	Incidence (% in Parenthesis)	LBW<br>Crude OR (95% CI in Parenthesis)
Total	398120 (100.0)	23103 (5.8)	
**Infant Sex**			
Male	203784 (51.2)	10911 (5.35)	0.85 (0.83−0.87)
Female	194336 (48.8)	12192 (6.27)	1
**Year of Birth**			
2000	25059 (6.3)	1833 (7.31)	1.27 (1.20−1.34)
2001	99235 (24.9)	5588 (5.63)	0.96 (0.92−1.00)
2002	94142 (23.7)	5281 (5.61)	0.95 (0.92−0.99)
2003	92050 (23.1)	5262 (5.72)	0.97 (0.94−1.01)
2004	87634 (22.0)	5139 (5.86)	1
**Season of Birth**			
Fall	108281 (27.2)	6362 (5.88)	1
Winter	97331 (24.5)	5344 (5.66)	0.96 (0.93−1.00)
Spring	94456 (23.7)	5600 (5.71)	0.97 (0.94−1.01)
Summer	98052 (24.6)	5797 (5.96)	1.01 (0.98−1.05)
**Material Race/Ethnicity**			
Non−Hispanic White (reference)	160133 (42.70)	6467 (4.04)	1
Non−Hispanic Black	74808 (19.95)	9163 (12.25)	3.03 ± 0.100
Hispanic	119215 (31.79)	6039 (5.07)	1.25 ± 0.045
Non−Hispanic Other	20861 (5.56)	1434 (6.87)	1.70 ± 0.100

**Maternal Age at Birth (years)**			
18 or younger	27676 (7.0)	2591 (9.36)	1
19–25	120821 (30.4)	7627 (6.31)	0.65 (0.62–0.68)
26–34	187853 (47.2)	9229 (4.91)	0.5 (0.48–0.52)
35 or older	61770 (15.5)	3656 (5.92)	0.61 (0.58–0.64)
**Maternal Weight Gain**			
Less than 25 lbs. gained	111361 (28.0)	10900 (9.40)	1.9 (1.84–1.95)
25–35 lbs. gained	160170 (40.2)	8304 (5.18)	1
More than 35 lbs. gained	121985 (30.6)	3899 (3.20)	0.6 (0.58–0.63)
**Maternal Education**			
Less than high school	96248 (24.2)	6753 (7.02)	1
High school graduate	102780 (25.8)	6813 (6.63)	0.94 (0.91–0.97)
Some college	77039 (19.6)	4623 (6.00)	0.85 (0.82–0.88)
College graduate or more	122053 (30.7)	4914 (4.03)	0.56 (0.54–0.58)
**Maternal Country of Birth**			
United States	265474 (66.7)	16915 (6.37)	1
Other Country	132646 (33.3)	6188 (4.67)	0.72 (0.70–0.74)
**Marital Status**			
Married	254340 (63.9)	11014 (4.33)	1
Unmarried	143780 (36.1)	12089 (8.41)	2.03 (1.98–2.08)
**Parity**			
First birth	138943 (34.9)	9094 (6.55)	1
One or more children	259177 (65.1)	14009 (5.41)	0.82 (0.80–0.84)
**Pregnancy Interval**			
First birth	138943 (34.9)	9094 (6.55)	1
Less than 1 year	7334 (1.8)	900 (12.27)	2 (1.85–2.15)
1 year or more	251843 (63.3)	13109 (5.21)	0.78 (0.76–0.80)
**Prenatal Care (Modified Kessner Index)**			
Adequate	297466 (74.7)	15040 (5.06)	1
Intermediate	72402 (18.2)	5084 (7.02)	1.42 (1.37–1.47)
Inadequate	28252 (7.1)	2979 (10.54)	2.21 (2.12–2.30)
**Maternal Smoking During Pregnancy**			
No	375001 (94.2)	20157 (5.38)	1
Yes	23119 (5.8)	2946 (12.74)	2.57 (2.46–2.68)
**Maternal Alcohol Consumption During Pregnancy**			
No	396301 (99.5)	22846 (5.76)	1
Yes	1819 (0.5)	257 (14.13)	2.69 (2.65–2.73)
**Delivery Method**			
Vaginal	307275 (77.2)	15551 (5.06)	1
Primary C–Section	53698 (13.5)	5350 (9.96)	2.08 (2.01–2.15)
Repeat C–Section	37147 (9.3)	2202 (5.93)	1.18 (1.13–2.23)
**Maternal Medical**			
None	287689 (72.3)	13230 (4.60)	1
At least one	110431 (27.7)	9873 (8.94)	2.04 (1.98–2.10)
**Congenital Anomalies**			
None	393034 (98.7)	22456 (5.71)	1
At least one	5086 (1.3)	647 (12.72)	2.41 (2.21–2.61)
**Maternal Residence at Time of Birth**			
Not City of Chicago	263730 (59.5)	11235 (4.75)	1
City of Chicago	161390 (40.5)	11868 (7.35)	1.59 (1.55–1.64)

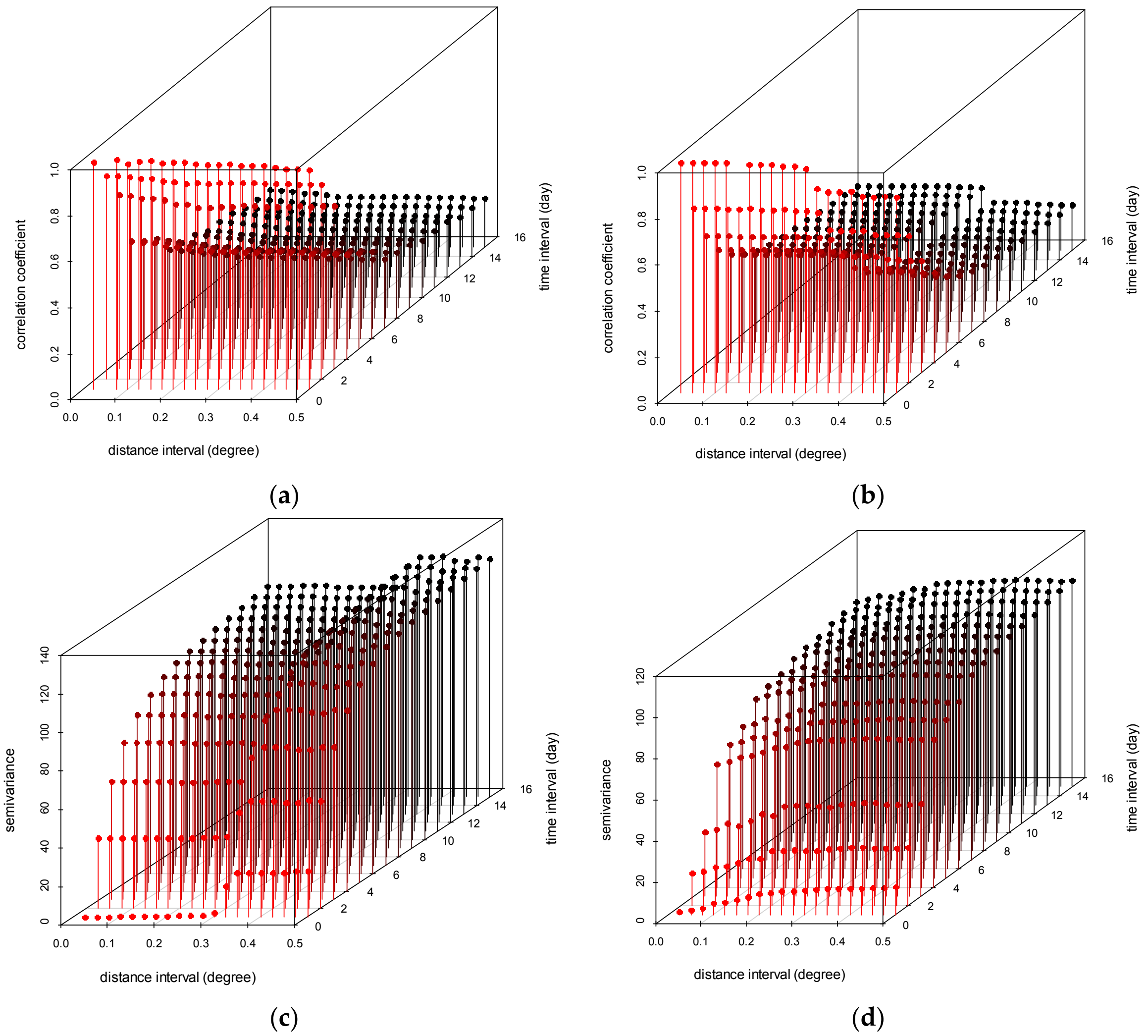

Figure S1. Spatiotemporal autocorrelation and semivariance of $PM_{2.5}$: (**a**) autocorrelation Illinois; (**b**) autocorrelation Ohio; (**c**) semivariance, Illinois; (**d**) semivariance, Ohio.

© 2016 by the authors. Submitted for possible open access publication under the terms and conditions of the Creative Commons Attribution (CC-BY) license (http://creativecommons.org/licenses/by/4.0/).
